# Non-thermal plasma efficiently decontaminates sensitive materials from respiratory viruses

**DOI:** 10.3389/fmicb.2026.1897956

**Published:** 2026-09-24

**Authors:** Chantal Lucini, Klára Obrová, Eva Vaňková, Josef Khun, Jan Hodek, Lucie Ulrychová, Jan Weber, Radek Přikryl, Lukáš Kalina, Štěpán Krobot, Vladimír Scholtz, Thomas Lion

**Affiliations:** 1 St. Anna Children’s Cancer Research Institute (CCRI), Vienna, Austria; 2 Ludwig Boltzmann Institute for Hematology and Oncology, Medical University of Vienna, Vienna, Austria; 3 Department of Physics and Measurements, University of Chemistry and Technology, Prague, Czechia; 4 Department of Biotechnology, University of Chemistry and Technology, Prague, Czechia; 5 Institute of Organic Chemistry and Biochemistry of the Czech Academy of Sciences, Prague, Czechia; 6 Department of Genetics and Microbiology, Faculty of Sciences, Charles University, Prague, Czechia; 7 Faculty of Chemistry, Brno University of Technology, Brno, Czechia; 8 Department of Pediatrics, Medical University of Vienna, Vienna, Austria

**Keywords:** adenovirus, cold atmospheric plasma, disinfection, human respiratory viruses, influenza A, rhinovirus, SARS-CoV-2

## Abstract

**Introduction:**

Heat- and moisture-sensitive porous materials such as plush toys, paper products, and foam are widely used in pediatric and healthcare environments but cannot be decontaminated by conventional liquid disinfectants, heat, or UV-based methods. This creates a need for alternative dry, non-thermal technologies. Non-thermal plasma (NTP) offers a promising approach for disinfecting such materials without compromising their integrity.

**Methods:**

In this study, we assessed the virucidal efficacy of a prototyped NTP device on four clinically relevant respiratory viruses—Severe Acute Respiratory Syndrome Coronavirus 2 (SARS-CoV-2), Influenza A virus (IAV), human Adenovirus (HAdV), and human Rhinovirus (HRV)—applied to paper, plush fabric, and foam rubber.

**Results:**

Material characterization by SEM and XPS demonstrated only mild surface oxidation and no structural damage following NTP exposure. Across all substrates, NTP treatment produced time-dependent reductions in viral infectivity. Maximum reductions ranged from 5.5–6.3 log_10_ for SARS-CoV-2, 6.3–7.1 log_10_ for IAV, 4.0–5.9 log_10_ for HAdV, and 2.7–5.0 log_10_ for HRV, depending on the virus-material combination. Enveloped viruses (SARS-CoV-2 and IAV) were inactivated more rapidly than the non-enveloped viruses HAdV and HRV, although all viruses were reduced to nearly or completely undetectable levels after 30–60 min of treatment. Several virus-material combinations exhibited biphasic inactivation kinetics characterized by an initial rapid decline followed by a slower phase of inactivation.

**Discussion:**

These findings support that NTP could be an effective, residue-free, and material-preserving method for inactivating a broad spectrum of respiratory viruses on porous substrates incompatible with conventional disinfection. NTP thus might represent a valuable tool for safely reusing sensitive items in pediatric and clinical settings and reducing contamination risks where existing methods are impractical.

## 1 Introduction

Respiratory viruses are major global pathogens because of their high transmissibility, ability to persist on surfaces, and potential to cause epidemics. Although airborne spread is the principal route of transmission, contaminated surfaces and shared objects represent important secondary reservoirs, particularly in communal and healthcare settings (Haas et al., 2023). This risk is especially pronounced in hospitals, daycare centers, and pediatric wards, where high-touch items are frequently reused without adequate decontamination between uses. To address this challenge, the present study examines the virucidal efficacy of non-thermal plasma (NTP), a dry, low-temperature disinfection technology, against four clinically relevant respiratory viruses with distinct biological properties: severe acute respiratory syndrome coronavirus 2 (SARS-CoV-2), influenza A virus (IAV), human rhinovirus (HRV), and human adenovirus (HAdV).

SARS-CoV-2, an enveloped RNA virus, demonstrated remarkable epidemic potential during the recent pandemic (Fathizadeh et al., 2020). Although enveloped viruses are generally more susceptible to disinfectants, the prolonged stability of SARS-CoV-2 on surfaces and in aerosols underscores the need for reliable decontamination strategies (van Doremalen et al., 2020). Moreover, repeated outbreaks caused by related coronaviruses, including SARS-CoV and MERS-CoV, indicate that members of the Coronaviridae family are likely to remain relevant targets for future infection-control efforts (Civljak et al., 2020). IAV, another enveloped RNA virus, is a frequent cause of seasonal and localized epidemic outbreaks (Moa et al., 2001). Its persistence in aerosols, low infectious dose, and rapid antigenic evolution make it a continuing public health challenge and a priority target for improved disinfection approaches (Tellier, 2009; Wu et al., 2020).

Non-enveloped viruses such as HRV and HAdV present additional challenges because of their marked environmental stability. HRV, a ubiquitous cause of respiratory tract infections, can remain infectious on surfaces and is difficult to eliminate from porous substrates because of its small particle size and structural resilience. HAdV is among the most resistant human viruses; its robust non-enveloped capsid and stable DNA genome make it particularly difficult to inactivate and therefore a useful indicator organism for evaluating disinfection technologies (Lion, 2014; Lion, 2019). Although adenovirus infections are often self-limiting in immunocompetent individuals, they can cause severe outbreaks in crowded settings and life-threatening disease in immunocompromised patients (Hiwarkar et al., 2018; Sammons et al., 2019).

Standard decontamination approaches, including ultraviolet (UV) irradiation, gamma irradiation, and chemical disinfectants, are often poorly suited to sensitive materials. UV light has limited penetration, and its efficacy is substantially reduced by surface irregularities and shadowing (Deng et al., 2020; Derraik et al., 2020; Kayani et al., 2021; Mills et al., 2018). Gamma irradiation requires specialized infrastructure and may compromise material integrity (Derraik et al., 2020; Rutala et al., 2023). Conventional liquid disinfectants are incompatible with many moisture- or heat-sensitive items and may leave residues that are undesirable in pediatric or clinical environments (Rutala et al., 2023; Obrova et al., 2022).

These limitations are particularly relevant for porous, soft, and absorbent materials such as plush toys, foam rubber, paper, and cardboard. Plush toys, often composed of polyester or cotton fibers with sponge-like inner structures, can trap microbes deep within multiple layers. Foam rubber, including polyurethane-based materials used in cushions, child seating, and packaging, readily absorbs fluids and resists complete drying. Paper and cardboard, which are common in books, educational toys, and packaging, may deform or degrade when exposed to heat or moisture (Rutala et al., 2023; Scholtz et al., 2023).

Such materials are ubiquitous in pediatric environments, where tactile play, frequent handling, and hand-to-mouth behavior increase the risk of contamination and transmission. Children are also more susceptible to infection because their immune systems are still developing, and viral shedding may be greater and more prolonged than in adults (Costa et al., 2012; Prendergast et al., 2012). Consistent with this vulnerability, contaminated toys and shared supplies in hospitals and daycare centers have repeatedly been implicated in outbreaks of influenza and adenovirus infections among children (Bearden and Casanova, 2016).

Against this background, NTP has emerged as a promising alternative disinfection technology. NTP is a partially ionized gas generated at room temperature and atmospheric pressure that contains reactive oxygen and nitrogen species (RONS), UV photons, ions, and electrons (Scholtz et al., 2023; Graves, 2012). In contrast to conventional methods, NTP provides dry, non-thermal, and non-wetting disinfection, making it particularly suitable for soft and porous materials that would otherwise need to be discarded after contamination (Obrova et al., 2022; Scholtz et al., 2023). Previous studies have shown that NTP can inactivate SARS-CoV-2, IAV, HRV, and HAdV on parafilm, P3 R filter material, foam, and other filter-based substrates, with dose-dependent reductions in viral titers (Obrova et al., 2022; Bouazizi et al., 2024).

A further advance has been the development of portable, affordable, 3D-printed plasma devices optimized for microbiocidal performance and improved surface coverage of irregular materials such as plush toys and layered foams. By promoting deeper penetration and more uniform exposure, these devices may enable the safe reuse of items in pediatric care while reducing waste, lowering transmission risk, and extending the availability of essential resources during periods of shortage, including pandemics (Obrova et al., 2022; Scholtz et al., 2023; Bouazizi et al., 2024; Klenivskyi et al., 2024).

## 2 Materials and methods

### 2.1 Non-thermal plasma (NTP) device

NTP was generated by point-to-ring corona discharge in a device whose operating parameters (discharge power: 0.7 W, voltage: 6–8 kV, current: 100–150 μA) were specifically optimized for enhanced antimicrobial and virucidal efficacy. The optimization process is described in detail in our previous works (Bouazizi et al., 2024; Klenivskyi et al., 2024), which involved systematic variation of the interelectrode distance (0–8 mm) and discharge current (50–500 μA) and assessment of the bactericidal effect on *Escherichia coli*. The selected parameters represent the optimal balance between maximum antimicrobial activity and stable, spark-free discharge operation.

Further details of the NTP device and the concentrations of long-lived plasma-generated species (NO_2_, N_2_O, O_3_, and H_2_O_2_) measured under identical operating conditions were described in our previous study (Bouazizi et al., 2024).

### 2.2 Assessment of material damage by NTP using scanning electron microscopy (SEM)

Material morphology and surface structure were examined by SEM using a Vega II LSH instrument (TESCAN GROUP, a.s., Brno, Czech Republic) to visualize potential NTP-induced alterations accurately. Small pieces were cut from the samples and mounted on a sample holder using double-sided conductive adhesive tape. All specimens were then sputter-coated with a thin gold layer under an argon atmosphere (15 mA, 60 s) using a POLARON Emitech SC7640 sputter coater (Quorum Technologies Ltd., Lewes, UK). Imaging parameters, including accelerating voltage (kV), working distance (WD; mm), and magnification, are provided at the bottom of the corresponding images in the Results section.

### 2.3 Chemical composition analysis of material surface using X-ray photoelectron spectroscopy (XPS)

X-ray photoelectron spectroscopy (XPS) was performed to characterize the chemical composition of the sample surfaces to a depth of ~5 nm using an Axis Ultra DLD spectrometer (Kratos Analytical Ltd., UK). A monochromatic Al Kα (hν = 1486.7 eV) X-ray source operating at 75 W (5 mA, 15 kV) was used, and spectra were collected over a 300 × 700 μm analytical area. High-resolution spectra were acquired with a step size of 0.1 eV and a pass energy of 20 eV. The Kratos charge neutralizer system was used for all analyses. The instrument base pressure during measurements was consistently 2 × 10^−8^ Pa. Spectra were analyzed using CasaXPS software (version 2.3.17) and charge-corrected to the main C 1 s carbon component (C–C, C–H), set to 285.00 eV. A standard Shirley background was used for all sample spectra.

As with the SEM analysis, XPS measurements were performed on samples exposed to NTP for 24 h—well beyond the antiviral treatment times—to evaluate the cumulative chemical effects of repeated disinfection cycles and to establish a safety margin for material integrity under extended plasma exposure.

### 2.4 Virus strains and cell lines

The following virus strains and cell lines were used: SARS-CoV-2 (hCoV-19/Czech Republic/NRL_6632_2/2020) (Bouazizi et al., 2024; Vankova et al., 2020), propagated in Vero E6 cells (ATCC CRL-1586) in DMEM containing Pen/Strep and 10% FBS; IAV H1N1/California/07/2009 (Diagnostic Hybrids, Athens, OH, United States), propagated in MDCK cells (ATCC CCL-34) in influenza growth medium (IGM) consisting of DMEM supplemented with Pen/Strep, 0.2% BSA, 1 mM HEPES, 13.6 mM L-glutamine, 42 mg/L DEAE-dextran, and 1 mg/L TPCK-trypsin; HAdV species C/type 2 (ATCC VR-846), propagated in A549 cells (DSMZ ACC107) in DMEM containing 10% FBS and Pen/Strep; and HRV A/type 2 (ATCC VR-482), propagated in HeLa Ohio cells (both a kind gift from G. ST Antivirals) in DMEM containing 10% FBS and Pen/Strep.

### 2.5 Material contamination and virus recovery

Paper, plush fabric, and foam rubber were selected as representative contaminated surfaces for evaluating NTP-mediated virus inactivation. Their suitability for direct pediatric contact after NTP treatment was not evaluated in this study. To assess whether the materials themselves affected viral infectivity or cell viability, we tested their intrinsic toxicity toward HRV and HAdV and toward HeLa and A549 cells (see Supplementary Materials). Virus recovery was performed according to the method published in the ISO Standard 18,184 (Textiles—Determination of antiviral activity of textile products, now referenced in the manuscript) (ISO, 2019a), as based on their characteristics, both plush and paper are similar to the fabrics covered by it. For plastic, repeated rinsing of the sample surface, as described in ISO 21702 (Measurement of antiviral activity on plastics and other non-porous surfaces) (ISO, 2019b), would have been sufficient. However, a uniform recovery method was used to ensure consistent handling of infectious material from all samples. Since RONS production is humidity-dependent relative humidity (RH) inside the device was measured after adding 6 mL of water to the cotton wool in the Petri dish, under the same conditions as during antiviral testing, during NTP treatment using a humidity sensor. For this purpose, measurements were performed with a testo 625instrument (Testo SE & Co. KGaA, Titisee-Neustadt, Germany) equipped with RH and temperature. The application range for RH measurement is 0–99.9 %RH, resolution is 0.1 %RH, accuracy 3 %RH in measuring in a range from 5 to 95 %RH. The temperature measuring range is −10–60 °C, resolution 0.1 °C, accuracy 0.5 °C.

#### 2.5.1 Paper

UV-treated Whatman I paper squares were contaminated with 30 μL of virus suspension in culture medium at a median tissue culture infectious dose of 10^6^ infectious units (IU)/mL, which soaked into the material. Drying led to a rapid loss of detectable infectivity even in the absence of NTP treatment, making a dried-inoculum approach unsuitable for assessing plasma-mediated inactivation. The squares were placed in the NTP device, and humidity was increased by adding a UV-sterilized cotton ball approximately 4 cm in diameter and wetted with 6 mL of sterile distilled water. A set of control samples was placed in another device that did not produce NTP. NTP was applied for 5, 10, 15, 20, and 60 min. Subsequently, residual virus was recovered by soaking the paper squares in 1 mL of DMEM without additives and vortexing thoroughly five times for 5 s each.

#### 2.5.2 Plush fabric

UV-treated squares of plush fabric were contaminated with 30 μL of virus suspension in culture medium at a median tissue culture infectious dose of 10^6^ IU/mL, applied as 10 droplets, each containing 3 μL of solution. The droplets were completely dried in a biosafety cabinet for 110 min, after which the squares were placed in the NTP device. A set of control samples was placed in another device that did not produce NTP. Humidity was increased by adding a UV-sterilized cotton ball approximately 4 cm in diameter and wetted with 6 mL of sterile distilled water. NTP was applied for 5, 10, 15, 20, and 60 min. Subsequently, residual virus was recovered by soaking the plush fabric squares in 1 mL of DMEM without additives and vortexing thoroughly five times for 5 s each.

#### 2.5.3 Foam rubber

UV-treated foam rubber circles were contaminated with 30 μL of virus suspension in culture medium at a median tissue culture infectious dose of 10^6^ IU/mL, applied as 10 droplets of 3 μL each. After the material had soaked for 15 min, the circles were placed in the NTP device. Drying led to a rapid loss of detectable infectivity even in the absence of NTP treatment, making a dried-inoculum approach unsuitable for assessing plasma-mediated inactivation. A set of control samples was placed in another device that did not produce NTP. Humidity was increased by adding a UV-sterilized cotton ball approximately 4 cm in diameter and wetted with 6 mL of sterile distilled water. NTP was applied for 5, 10, 15, 20, and 60 min. Subsequently, residual virus was recovered by soaking the foam rubber circles in 1 mL of DMEM without additives and vortexing thoroughly five times for 5 s each.

### 2.6 Virus infectious titer determination

The recovered suspensions were used directly to infect the permissive cells specified above and determine the infectious titer of each virus according to the respective protocols described below. SARS-CoV-2 was titrated by plaque assay using 1:10 serial dilutions to infect Vero E6 cells (250,000 cells/well) in 24-well plates. Infected Vero E6 cells were incubated at 37 °C in a 5% CO_2_ incubator for 4 h. Subsequently, the inoculum was overlaid with 3% carboxymethyl cellulose in DMEM/PenStrep/10% FBS and incubated for 5 days at 37 °C and 5% CO_2_. IAV was titrated by plaque assay using 1:10 serial dilutions. Virus was transferred onto MDCK cells (180,000 cells/well, plated the day before the experiment) in 24-well plates. After 1 h of incubation, the cells were washed once with PBS, 400 μL of IGM was added, and the medium was overlaid with 400 μL of 1.2% carboxymethyl cellulose in IGM without TPCK-trypsin. The cells were then incubated for 2 days at 37 °C and 5% CO_2_. For both SARS-CoV-2 and IAV plaque assays, infection was terminated by aspirating the medium. The cells were washed once with PBS, fixed, and stained with naphthalene black. After 45 min, the staining solution was aspirated, the cells were washed with water, and the resulting plaque-forming units (PFU) were counted. Viral titers were expressed as PFU/mL. HAdV was titrated by a TCID_50_ assay using 1:10 serial dilutions to infect A549 cells in 96-well plates. The cells were incubated at 37 °C in a CO_2_ incubator for 7 days, and the cytopathic effect (CPE) was quantified using the crystal violet assay, as described previously (Lee et al., 2015). HRV was titrated by a TCID_50_ assay using 1:10 serial dilutions to infect HeLa Ohio cells in 96-well plates. The cells were incubated at 37 °C in a CO_2_ incubator for 5 days, and the CPE was quantified using the crystal violet assay, as described previously (Lee et al., 2015). The respective infectious titers, expressed in IU/mL, were calculated using the Spearman–Kärber method (Kärber, 1931).

### 2.7 Statistical analysis

All data were generated from three biological replicates and analyzed using the Shapiro–Wilk test to assess data distribution. A *p*-value > 0.05 indicated that the data followed a normal distribution. A two-sided t-test comparing treated and control samples at each time point was therefore used to determine the significance of differences. Results with *p*-values < 0.05 were considered significant and are presented as the mean ± standard deviation. Additionally, the log_10_ reduction was reported as the primary effect size, with paired Cohen’s dz. included as an exploratory standardized effect when all six replicate titers were positive numerical values and the paired log differences had non-zero variance. Replicate-level measurements, measures of variability, raw *p*-values, and Benjamini–Hochberg (BH)-adjusted q-values were reported. To control the false discovery rate (FDR), all 66 raw *p*-values from the four viruses, three materials, and five to six exposure times were treated as one prespecified hypothesis family. Statistical significance after correction was defined as q < 0.05. No prospective power analysis was performed before the experiment; the additional power calculations are retrospective planning aids based on an explicit BH approximation and should not be interpreted as validating non-significant results (see Supplementary Table).

## 3 Results

### 3.1 Non-thermal plasma has limited impact on sensitive materials

To assess the impact of NTP on the structural integrity of sensitive materials, SEM imaging was performed on untreated and NTP-treated samples. Microscopic examination of untreated and NTP-treated samples revealed distinct material-specific behaviors. The paper substrate showed no substantial morphological alterations following NTP exposure when comparing the reference sample (Figure 1A) with the sample after 24 h of NTP treatment (Figure 1B). Plush fabric fibers maintained a smooth surface devoid of defects, fractures, or adherent particles. However, a slight increase in fiber coiling and twisting was observed, which may be related to the localized effects of NTP on surface tension or structural elasticity (Figures 1C,D). In contrast, the foam rubber sample exhibited inherently high porosity, with numerous micrometer-scale particles uniformly distributed across its surface (Figure 1F). NTP treatment did not induce discernible changes in the morphology or abundance of these particles, suggesting limited susceptibility to surface modifications under the applied NTP conditions (Figure 1E). In summary, paper showed no detectable structural changes, plush fabric exhibited only minor alterations, and foam rubber maintained its structural integrity after treatment.

**Figure 1:**
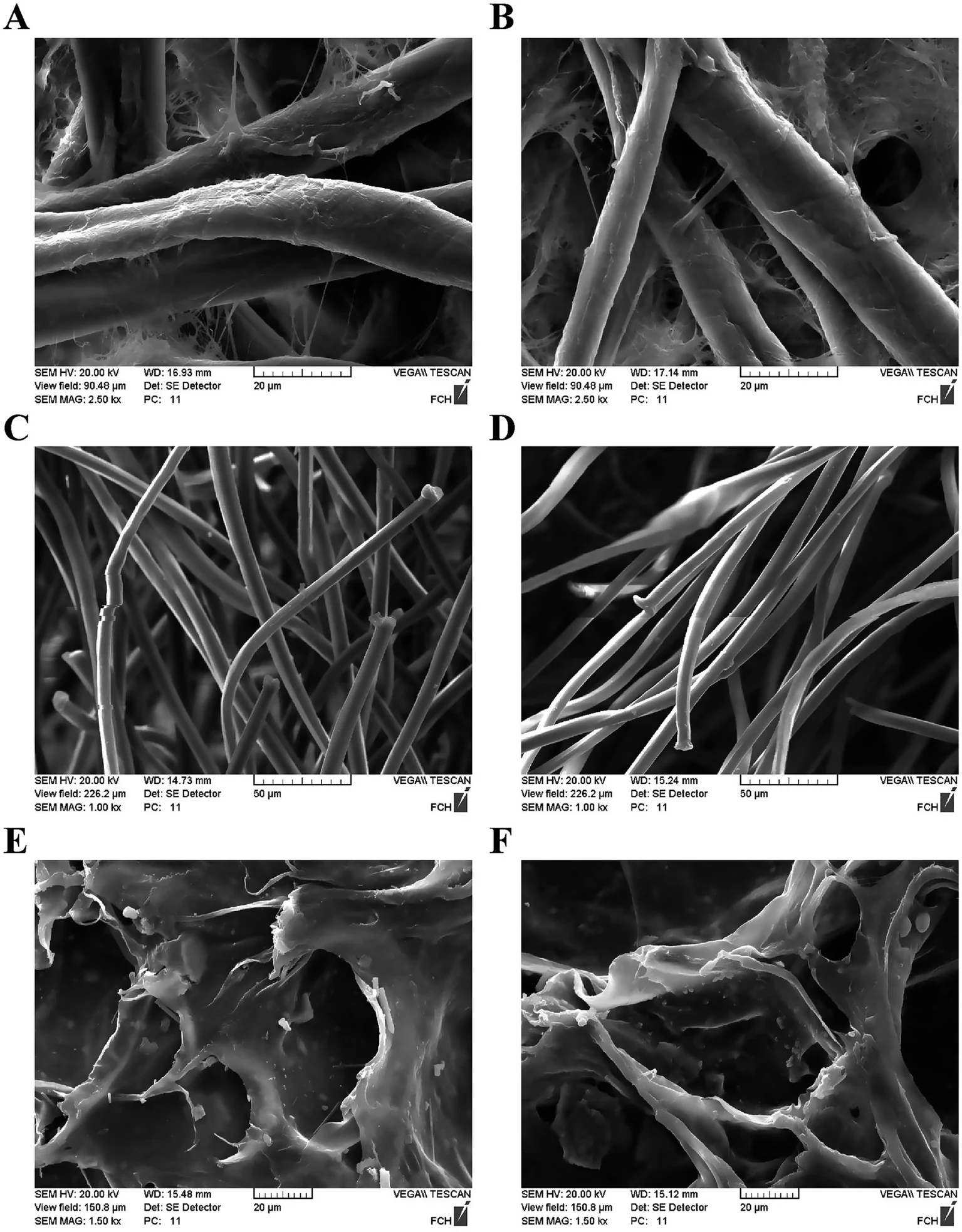
SEM images of untreated and NTP-treated materials **(A)** NTP-untreated paper at 2500 × magnification; **(B)** paper after 24 h of NTP at 2500 × magnification; **(C)** NTP-untreated plush fabric at 1000 × magnification; **(D)** plush fabric after 24 h of NTP exposure at 1000 × magnification; **(E)** NTP-untreated foam rubber at 1500 × magnification; **(F)** foam rubber after 24 h of NTP exposure at 1500 × magnification. SEM: scanning electron microscopy, NTP: non-thermal plasma.

### 3.2 Superficial oxidation of the material surfaces exposed to NTP analyzed by X-ray photoelectron spectroscopy (XPS)

#### 3.2.1 Paper

XPS analysis was performed to quantify the surface chemical modifications induced by plasma treatment. The elemental composition of the untreated reference sample was 58.7 at.% carbon and 41.3 at.% oxygen. Following 24 h of NTP exposure, a clear shift in surface chemistry was observed: the carbon content decreased to 55.5 at.%, while the oxygen concentration increased to 42.9 at.% (Figures 2A,B). This increase in oxygen content is consistent with the surface oxidation of cellulose occurring during air plasma discharge. Additionally, the analysis detected the incorporation of 1.6 at.% nitrogen in the samples treated for 24 h (Figure 2B).

**Figure 2:**
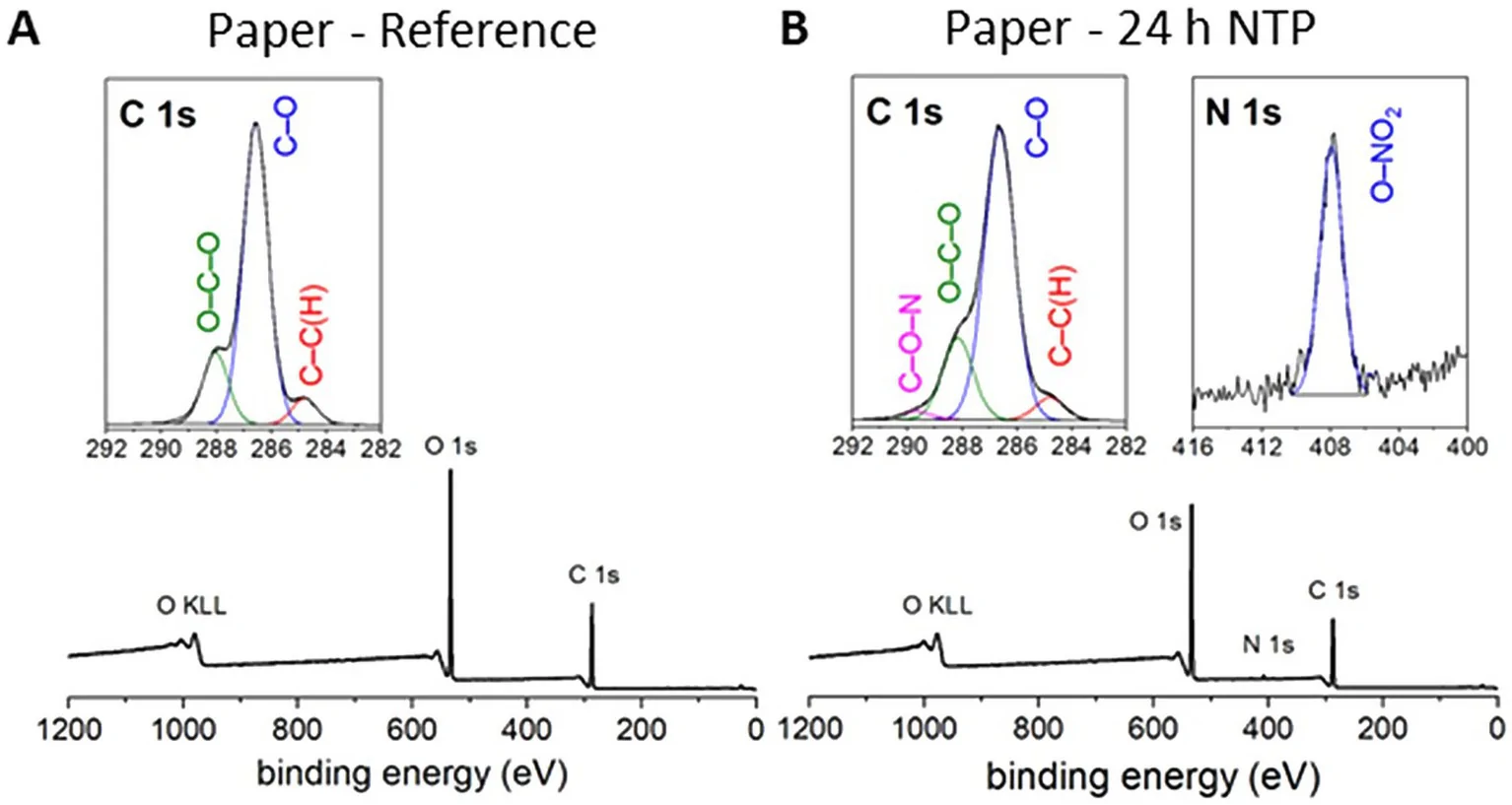
Spectra of chemical composition analysis of paper surface, **(A)** unexposed to NTP, **(B)** after 24 h of NTP exposure. Ref: reference, NTP: non-thermal plasma.

The high-resolution C 1 s spectra (Figures 2A,B) were resolved into components corresponding to C-C/C-H (284.8 eV), C-O (~286.6 eV), and O-C-O (~288.1 eV). Furthermore, a distinct signal identified at 289.7 eV was attributed to carbon atoms bonded to nitrooxy groups (-O-NO_2_). The formation of these organic functionalities was further supported by the high-resolution N 1 s spectra (Figure 2B), which exhibited a prominent peak at 408.0 eV, characteristic of nitrogen in a highly oxidized state within nitrooxy structures.

#### 3.2.2 Plush fabric

Polyester plush fabric exhibited progressive surface oxidation with increasing NTP exposure duration. The elemental composition of the untreated reference sample was 68.9 at.% carbon, 22.6 at.% oxygen, and 8.5 at.% silicon. Following 24 h of NTP exposure, the carbon content decreased to 64.0 at.%, while the oxygen concentration increased to 26.1 at.% (Figures 3A,B). Additionally, the silicon concentration rose to 9.9 at.%, likely due to plasma-induced etching of surface contaminants and subsequent exposure of the silanized layer.

**Figure 3:**
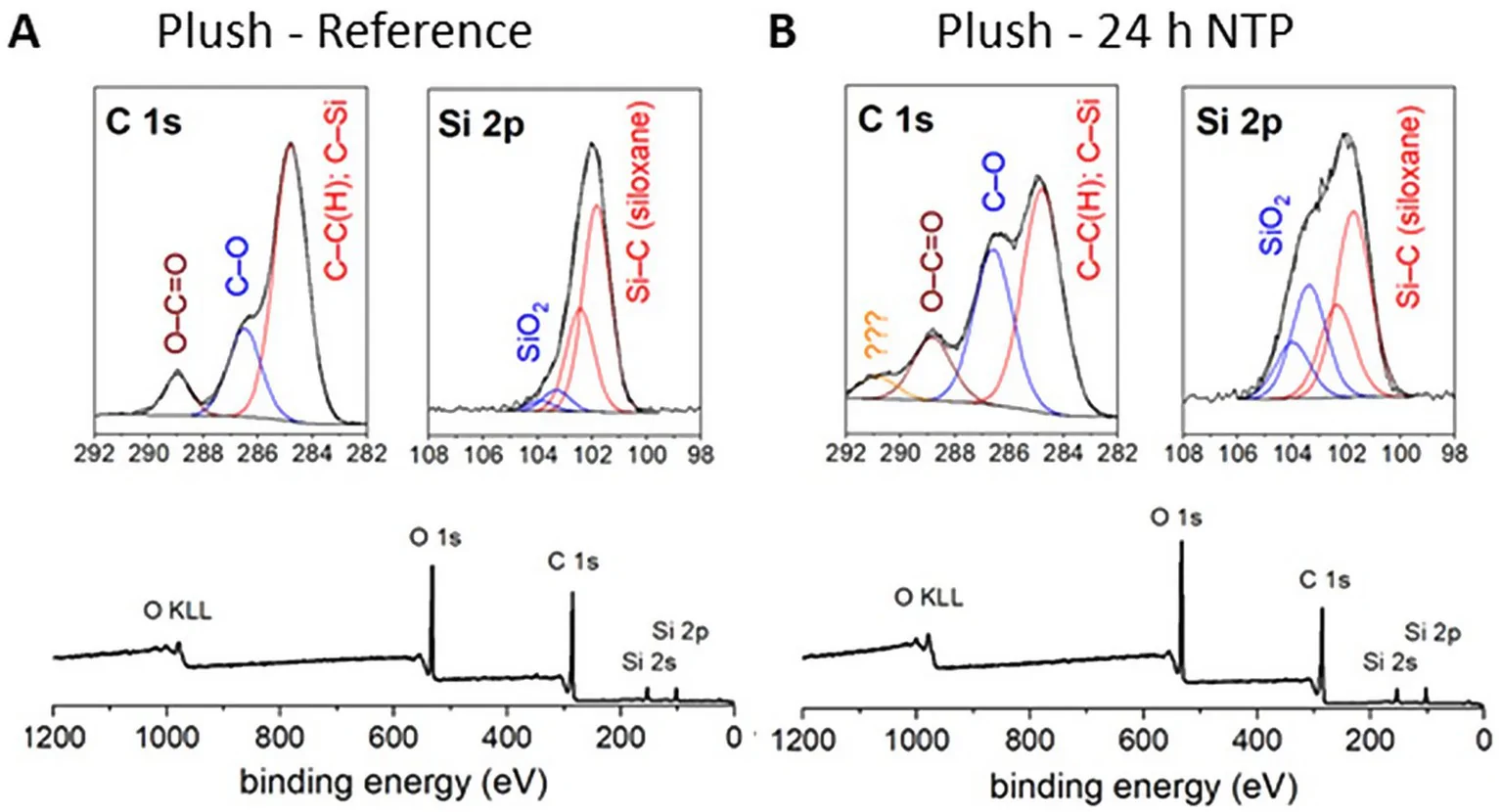
Spectra of chemical composition analysis of plush fabric surface, **(A)** unexposed to NTP, **(B)** after 24 h of NTP exposure. Ref: reference, NTP: non-thermal plasma.

High-resolution C 1 s spectra revealed that the intensity of the convoluted peak at 284.8 eV (C-C/C-H and C-Si) decreased after treatment. This change was accompanied by a marked increase in C-O bonds (~286.6 eV) and O-C=O functionalities (~288.8 eV), as well as the emergence of a new component at ~290.8 eV corresponding to carbonate species (CO_3_^2−^). Alongside this extensive oxidation of the organic matrix, a substantial transformation of the silicon-based additives was also observed. The Si 2p spectra showed a significant binding-energy shift of Si 2p3/2 from ~101.8 eV (organic silicon) to ~103.3 eV, characteristic of SiO_2_-like structures. These findings demonstrate that NTP exposure induces a dual modification consisting of extensive oxidation of the polyester backbone and mineralization of the organosilicon surface agents.

#### 3.2.3 Foam rubber

Although the precise chemical composition of the foam rubber was unavailable, XPS analysis revealed pronounced oxidative changes following NTP treatment. The elemental composition of the untreated reference sample was 95.9 at.% carbon, 3.3 at.% oxygen, 0.6 at.% nitrogen, and a small amount of zinc (0.2 at.%), the latter likely originating from ZnO used as a vulcanization activator. Following NTP exposure, the carbon content decreased to 85.4 at.%, while the oxygen and nitrogen concentrations increased to 11.7 at.% and 2.7 at.%, respectively (Figures 4A,B).

**Figure 4:**
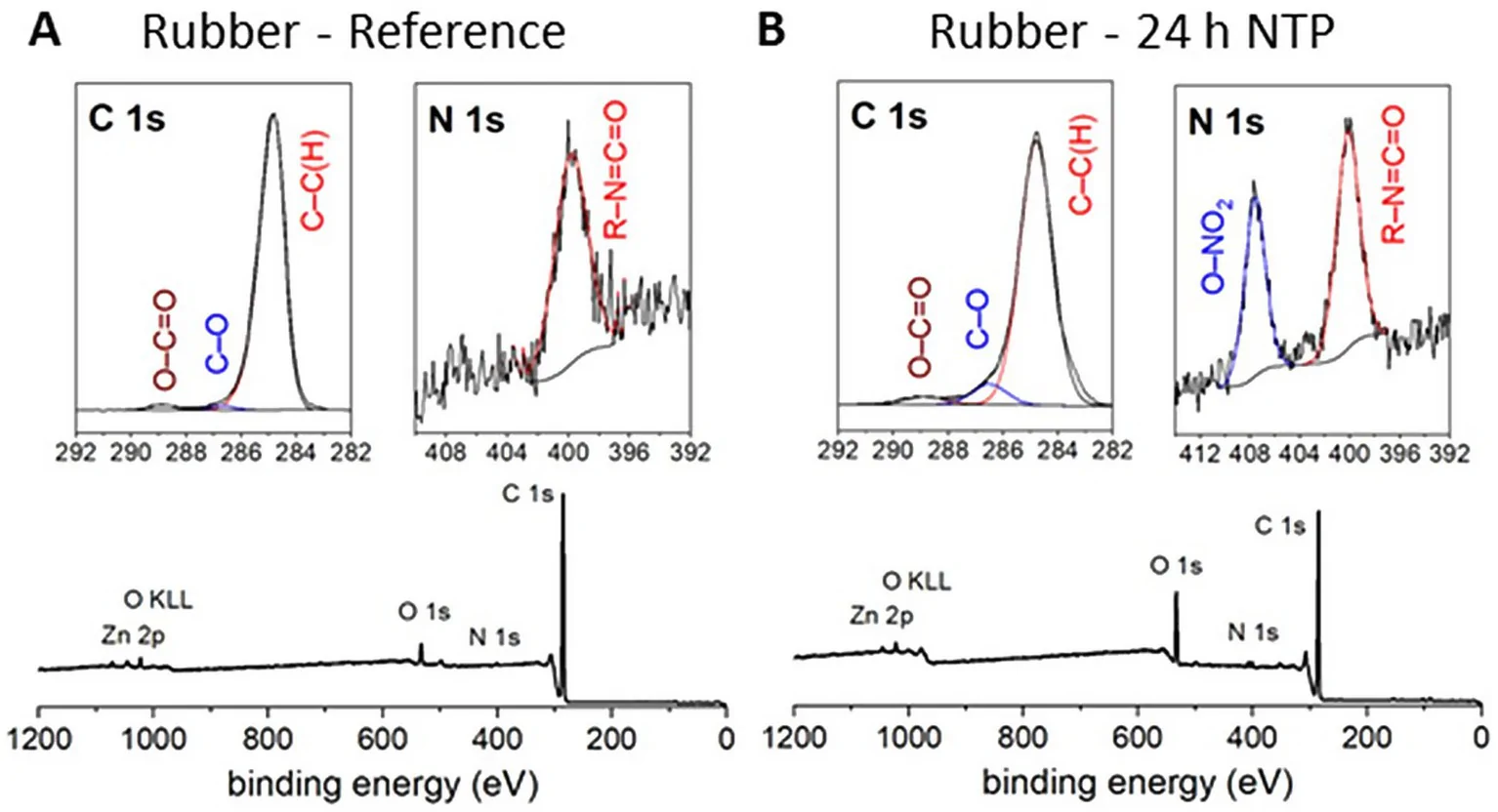
Spectra of chemical composition analysis of foam rubber surface, **(A)** unexposed to NTP, **(B)** after 24 h of NTP exposure. Ref: reference, NTP: non-thermal plasma.

High-resolution N 1 s spectra provided further insight into these changes. In the untreated reference sample, nitrogen was present predominantly at 399.9 eV, corresponding to isocyanate groups (R-N=C=O) or other organic nitrogen-containing additives. After NTP treatment, a new prominent peak emerged at 407.6 eV, characteristic of nitrogen in a highly oxidized state, specifically nitrate-like species or nitrooxy groups (-O-NO_2_). Alongside these nitrogen-related modifications, broader oxidation of the polymer backbone was evident in the high-resolution C 1 s spectra. The analysis revealed a substantial decrease in non-polar C-C/C-H bonds and a marked increase in oxidized functionalities, predominantly C-O bonds (~286.5 eV). Notably, unlike the cellulose samples, the C 1 s spectra of the foam did not exhibit a distinct resolved peak corresponding to carbon bonded within nitrooxy environments (C-O-N). This absence is likely explained by the low total nitrogen concentration (2.7 at.%), which renders the corresponding shifted carbon signal too weak to be reliably detected. Furthermore, any minor contribution from these nitrogen-bonded carbon species is likely obscured by partial overlap with the O-C=O (carboxyl/ester) component in the high-binding-energy region. Nevertheless, the pronounced overall increase in oxidized carbon bonds confirms extensive oxidative modification of the foam matrix induced by the NTP discharge.

### 3.3 Non-thermal plasma efficiently reduces viral infectivity on sensitive materials

Since none of the tested materials exhibited intrinsic cytotoxic or virucidal activity, the antiviral effects observed could be attributed solely to NTP exposure (see Supplementary Materials). The RH was 39.4% at 23.7 °C before NTP exposure, 93.7% at 25.0 °C after 5 min, 98.3% at 26.3 °C after 15 min, and 99.2% at 26.8 °C after 1 h.

To evaluate the antiviral efficacy of NTP, paper, plush fabric, and foam rubber were artificially contaminated with SARS-CoV-2, IAV, HAdV, or HRV and exposed to NTP for 5–60 min. Residual infectivity relative to untreated controls was quantified for each material-virus combination. The absolute viral titers, all log_10_ reduction (primary effect size) and all other statistical parameters described in the Methods are shown in the Supplementary Table.

SARS-CoV-2 inactivation displayed strong material dependence. On paper, infectivity first decreased significantly at 10 min (30.9 ± 15.3%, *p* ≈ 0.028), fell to 1.8 ± 1.2% by 20 min (*p* ≈ 0.0042), and no viral activity was detected after 30 min (0.00 ± 0.00%, *p* ≈ 0.0034). However, the highest log_10_ reduction (5.46) was observed after 60 min. Plush fabric showed a faster decline, with a significant reduction at 5 min (20.7 ± 5.9%, *p* ≈ 0.022) and nearly no detectable viral activity after 30 min (0.37 ± 0.27%, *p* ≈ 0.020), followed by no detectable infectivity after 60 min (0.00 ± 0.00%, *p* ≈ 0.0015). The highest log_10_ reduction (6.06) was also observed after 60 min. Foam rubber showed a rapid initial decline, with infectivity reduced to 48.4 ± 0.56% (*p* ≈ 0.00010) at 5 min and below 15% by 10 min (13.0 ± 0.85%, *p* ≈ 0.00018). No detectable viral activity was observed after 40 min (0.00 ± 0.00%, *p* ≈ 0.0026) and log_10_ reduction was almost the same between 30 and 60 min (6.25 and 6.26; Figure 5A, Supplementary Table).

**Figure 5:**
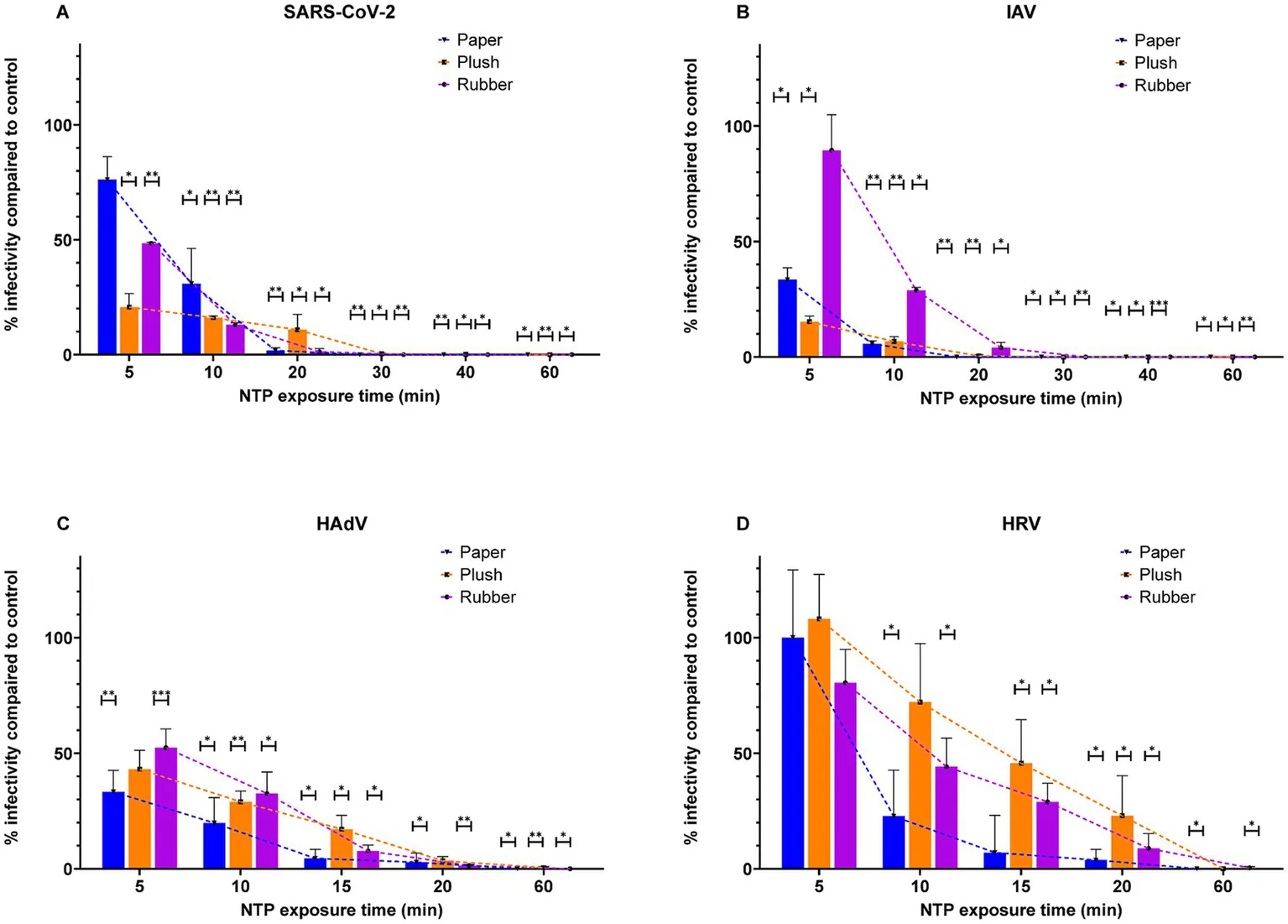
**(A–D)** Reduction in infectivity of the selected viruses following exposure of sensitive materials (paper, plush fabric, and foam rubber) to NTP. Data are expressed as the percentage of untreated controls (samples exposed to identical conditions without NTP treatment). Values represent the mean ± SD of three independent biological replicates. Statistical significance was determined using a two-sided t-test comparing NTP-treated and control samples at each time point (*p* < 0.05, *p < 0.005, **p < 0.0005). Absolute viral titers are provided in Supplementary Table. NTP treatment resulted in a time-dependent reduction in infectivity for all viruses, with infectious titers reaching near-undetectable or undetectable levels after 20–60 min, depending on the virus-material combination. HAdV = human adenovirus; HRV = human rhinovirus; IAV = influenza A virus; SARS-CoV-2 = severe acute respiratory syndrome coronavirus 2.

IAV infectivity declined rapidly on all materials. After 5 min of NTP exposure, paper and plush fabric showed strong reductions (33.5 ± 5.1%, *p* ≈ 0.019 and 15.1 ± 2.5%, *p* ≈ 0.0087, respectively). Foam rubber required 10 min to fall below 30% infectivity (28.9 ± 1.26%, *p* ≈ 0.0060). No detectable viral activity was observed after 30 min on paper and plush fabric (both 0.00 ± 0.00%, *p* ≈ 0.0064 and *p* ≈ 0.0051, respectively), and after 60 min on foam rubber (0.00 ± 0.00%, *p* ≈ 0.0045) (Figure 5B, Supplementary Table). The strongest log_10_ reduction in paper and plush was after 30 min (6.26 in paper and 7.11 in plush), whereas in rubber it was observed after 60 min (6.28).

HAdV exhibited slower inactivation on plush fabric than on paper or foam rubber. Paper and foam rubber showed significant reductions after 5 min (33.3 ± 9.3%, *p* ≈ 0.0035 and 52.4 ± 8.1%, *p* ≈ 0.0000093, respectively). Infectivity fell below 10% by 15 min (4.5 ± 3.9%, *p* ≈ 0.0089 in paper and 7.7 ± 2.5%, *p* ≈ 0.034 in foam rubber), and no detectable viral activity was observed at 60 min (both 0.00 ± 0.00%, *p* ≈ 0.050 and *p* ≈ 0.040, respectively). In both materials the largest log_10_ reduction was also observed after 60 min (3.98 and 5.85 respectively). Plush fabric showed its first significant reduction at 10 min (29.0 ± 4.6%, *p* ≈ 0.0014), with residual infectivity of 17.1 ± 6.0% at 15 min and nearly no detectable viral activity after 60 min (0.49 ± 0.39%, *p* ≈ 0.026) (Figure 5C, Supplementary Table). Also in this case the largest log_10_ reduction was observed after 60 min (4.76).

HRV also showed material-dependent inactivation kinetics. On paper, infectivity dropped significantly after 10 min (22.8 ± 19.9%, *p* ≈ 0.021), fell below 5% by 20 min (3.8 ± 4.6%, *p* ≈ 0.035), and no infectivity was detected after 60 min (0.00 ± 0.00%, *p* ≈ 0.040), when the largest log_10_ reduction was observed (4.10)- Plush fabric showed its first significant reduction only at 15 min (45.7 ± 18.8%, *p* ≈ 0.047), followed by a continued decline and no measurable viral activity at 60 min (0.00 ± 0.00%, *p* ≈ 0.090). Also in this case the largest log_10_ reduction was observed after 60 min (2.69). Foam rubber demonstrated a significant reduction at 10 min (44.2 ± 12.3%, *p* ≈ 0.030), fell below 10% by 20 min (8.8 ± 6.5%, *p* ≈ 0.032), and showed nearly no detectable viral activity after 60 min (0.38 ± 0.53%, *p* ≈ 0.026), when log10 reduction was at is highest (5.01) (Figure 5D, Supplementary Table).

Overall, NTP reduced the infectivity of all four respiratory viruses to nearly below or below the limit of detection across all materials tested, with inactivation kinetics dependent on both viral properties and material characteristics.

## 4 Discussion

This study supports that NTP is an efficient and material-compatible method for inactivating a broad spectrum of human respiratory viruses on porous, heat- and moisture-sensitive substrates. By evaluating four epidemiologically relevant viruses—SARS-CoV-2, IAV, HAdV and HRV—across three commonly used materials in pediatric and healthcare settings (paper, plush fabric, foam rubber), we showed that NTP consistently was able to reduce viral infectivity to at least nearly undetectable levels of infectivity after 30 to 60 min, depending on virus–material combinations. These findings expand on our previous work demonstrating the virucidal effects of NTP on surface contaminants, including SARS-CoV-2 and IAV on mask filters and other materials (Obrova et al., 2022; Bouazizi et al., 2024).

Material characterization provided essential insight into why all three substrates tolerated NTP exposure without major structural or chemical compromise. SEM analysis confirmed no change in structural attributes at all for paper, only minor changes for plush and foam rubber maintained its structure after treatment. XPS revealed mainly superficial oxidation—manifested as increased oxygen-containing functional groups—consistent with the mild oxidative surface modification described in prior NTP–material interaction studies (Obrova et al., 2022; Scholtz et al., 2023). Crucially, these observations were made after 24 h of continuous NTP exposure, substantially exceeding the antiviral treatment times (5–60 min), thereby providing a conservative assessment of plasma-induced surface alterations under prolonged exposure. Importantly, no detrimental alterations in material morphology were observed, supporting the suitability of NTP for items that cannot undergo heat-, moisture-, or solvent-based decontamination (Rutala et al., 2023). However, the present assessment was limited to surface morphology (SEM) and surface chemistry (XPS) and therefore could not provide a comprehensive evaluation of the mechanical performance or long-term durability of the tested materials. Regarding the durability of NTP-treated materials under realistic conditions, mechanical properties of paper exposed to NTP under comparable discharge parameters were previously evaluated and shown to remain unchanged even after 120 min of treatment (Scholtz et al., 2023). For plush fabric and foam rubber, our SEM and XPS analyses suggested that plasma treatment induces predominantly superficial surface modifications, which are unlikely to result in substantial changes in mechanical integrity. A comprehensive assessment of multi-cycle durability and mechanical properties across all tested materials is currently in progress and will be the subject of a future study.

The differences in susceptibility observed among the four viruses can by some extent be explained by their structural organization and the primary molecular targets of plasma-generated RONS (Mumtaz and Rana, 2026; Ahmed et al., 2025). The more pronounced initial reductions observed for SARS-CoV-2 and IAV are consistent with the high susceptibility of enveloped viruses to oxidative damage. Lipid peroxidation and modification of envelope glycoproteins can rapidly abolish infectivity even before substantial genome degradation occurs, leading to steep early declines in viable virus counts (Obrova et al., 2022; Mumtaz and Rana, 2026; Lan et al., 2024; Huang et al., 2025). SARS-CoV-2 was the most susceptible virus, which is consistent with its fragile lipid envelope and the critical role of the spike glycoprotein in host-cell attachment. Oxidative damage to membrane lipids and envelope proteins can rapidly abolish infectivity even before extensive genome degradation occurs. IAV, although also enveloped, exhibited slightly greater persistence under some conditions. This may reflect differences in envelope composition, virion morphology, and the organization of its surface glycoproteins, which can influence susceptibility to oxidative modification (Obrova et al., 2022; Mumtaz and Rana, 2026; Lan et al., 2024; Huang et al., 2025). In contrast, non-enveloped viruses generally depend on the accumulation of damage to capsid proteins and nucleic acids before infectivity is lost, which may result in a more gradual reduction profile (Lion, 2019; Lan et al., 2024; Huang et al., 2025; Birmpa et al., 2016). HAdV possesses a highly stable icosahedral protein capsid composed of tightly packed structural proteins that protect the viral genome from environmental stress. Effective inactivation therefore requires the accumulation of damage to capsid proteins and/or nucleic acids, resulting in slower loss of infectivity (Lion, 2019). HRV is likewise non-enveloped and contains a compact protein capsid that contributes to its well-established environmental stability (Wang et al., 2017; Reagan et al., 1981). However, its smaller particle size and less complex capsid architecture compared with adenovirus may facilitate access of reactive species to critical viral structures, potentially explaining why HRV appeared more susceptible than HAdV despite also being non-enveloped (Huang et al., 2025; Wang et al., 2017; Reagan et al., 1981). Collectively, the observed susceptibility ranking is consistent with a hierarchy in which viruses protected only by a lipid envelope are most readily inactivated, whereas viruses relying on highly stable protein capsids require more extensive molecular damage before infectivity is lost. Recent plasma-biological interface studies further indicate that plasma-generated RONS and charged particles can act synergistically on biomolecular targets by oxidizing lipids, proteins, and nucleic acids, while also inducing downstream biological responses such as oxidative stress and immune modulation, with the relative contribution of each pathway depending on virion structure (Mumtaz and Rana, 2026; Ahmed et al., 2025; Lan et al., 2024).

However, the dependence of inactivation kinetics seems to rely on both the material properties and virus type. Beyond the overall reduction in infectivity, the shape of the inactivation curves may provide additional insight into the underlying mechanisms of NTP-mediated viral inactivation. Several virus-material combinations exhibited biphasic kinetics characterized by a rapid initial decrease in infectious titer followed by a slower phase of inactivation. Such tailing behavior has frequently been reported during plasma-based microbial decontamination and is generally attributed to population heterogeneity and limitations in agent accessibility rather than changes in plasma activity itself (Mošovská et al., 2023; Zhang and Wang, 2025). RONS are expected to rapidly inactivate virions that are directly exposed at the material surface, resulting in an initial steep decline. The remaining infectious fraction may consist of particles embedded within surface irregularities, retained in pores or fiber networks, or protected by aggregation and local microenvironmental effects, thereby requiring longer exposure times to achieve non-detectable levels of infectivity (Bouazizi et al., 2024; Mumtaz and Rana, 2026; Huang et al., 2025; Birmpa et al., 2016; Mošovská et al., 2023; Zhang and Wang, 2025). Material-dependent differences further support the importance of physical accessibility during treatment. Because virus application and drying conditions had to be adapted to the absorbency and drying tolerance of each substrate, cross-substrate comparisons should be interpreted cautiously and mainly as indicators of material-dependent viral accessibility. The delayed inactivation of HAdV on plush fabric compared with paper and foam rubber suggests that the dense fibrous structure may partially shield virions from direct exposure to reactive species. Porous and textured materials can create protected microenvironments where local RONS concentrations are lower and diffusion is restricted, producing extended tailing phases despite continued plasma exposure (Lan et al., 2024; Mošovská et al., 2023; Zhang and Wang, 2025). Conversely, smoother and more exposed surfaces facilitate more uniform contact between virions and reactive species, resulting in faster and more nearly log-linear reductions. However, shielding effect of fiber density was not directly tested in this study and will be topic of future work.

To further contextualize the observed antiviral effects, it is important to consider the plasma-generated species responsible for viral inactivation. In our previously characterized identical discharge (power 0.7 W, voltage 6–8 kV, current 100–150 μA), we measured the concentrations of long-lived reactive species, including NO₂, N₂O, O₃ and H_2_O_2_ (Bouazizi et al., 2024). These species are known to contribute to oxidative stress in biological targets, and their presence provides indirect evidence for the generation of more transient but highly reactive short-lived species, such as hydroxyl radicals (•OH), singlet oxygen (^1^O₂), and peroxynitrite (ONOO^−^), which are typically formed through secondary reactions in the gas phase and at the gas–liquid interface (Graves, 2012; Lan et al., 2024). Direct measurement of these short-lived species is technically demanding and requires specialized equipment. Regarding penetration into porous substrates, the observed inactivation kinetics suggest material-dependent differences. The similar inactivation kinetics observed for paper, plush fabric, and foam rubber are consistent with the possibility that reactive species are capable of penetrating the cellulose fiber matrix. However, direct measurements of species penetration were not performed in this study. Taken together, these observations indicate that NTP-mediated viral inactivation is governed not only by intrinsic viral susceptibility but also by the accessibility of virions within the material matrix, highlighting the combined roles of virus structure and surface characteristics in determining inactivation kinetics.

From an implementation perspective, the treatment durations required for the more resistant virus–material combinations also warrant consideration. Although 30–60 min exposures may be acceptable for batch decontamination of reusable sensitive items outside urgent workflows, such durations could limit application in high-throughput or time-critical clinical settings. Practical deployment will therefore require balancing antiviral efficacy with operational feasibility, including optimization of device geometry, plasma dose, loading capacity, and workflow integration.

While the present findings establish NTP as a promising decontamination technology, several translational considerations remain. NTP generates reactive byproducts, and occupational safety limits would require attention in practice. Additionally, our controlled laboratory conditions do not fully replicate real-world contamination involving organic matter or biofilms, which may scavenge reactive species and reduce efficacy. Furthermore, the potential structural damage to materials subjected to long-term plasma treatment needs to be evaluated. These important aspects are beyond the scope of the current foundational study but represent critical directions for future investigation.

Furthermore, the study was limited to n = 3 biological replicates providing limited power and unstable variance estimates. However, control titers were stable across exposure times in 11 of 12 virus–material series; HRV on plush was the exception (SD ≈ 0.53 log_10_; range ≈ 1.27 log_10_). The observed variability, particularly for HRV on plush fabric, may be attributable to the heterogeneous and highly porous structure of the material, which can influence virus distribution, recovery efficiency, and local exposure to reactive plasma species. In addition, minor variations in drying, adsorption, and extraction of virus particles from fibrous substrates are inherent challenges in carrier-based virucidal assays and may contribute to increased assay variability. Despite this, the overall trend toward substantial NTP-mediated viral inactivation remained consistent, with the majority of comparisons remaining statistically significant after correction for multiple testing. Out of the 66 unique control-NTP comparisons 55 had a raw *p* < 0.05 and 50 a BH-adjusted q < 0.05 still indicating a clear trend toward the potential of NTP as a disinfection tool in this context.

Future work may focus on identifying which NTP-generated species are most responsible for viral inactivation on specific materials—employing targeted scavenger experiments or advanced spectroscopic techniques— optimizing device geometry further for complex objects and evaluating performance under real-world contamination scenarios. Nonetheless, the present results supports the potential usage of NTP as a highly effective, residue-free, and non-thermal decontamination method specifically suited for materials that cannot be disinfected by conventional methods such as chemical soaking, high-temperature treatment, or UV irradiation—methods that are often limited by material incompatibility, penetration issues, or harmful by-products (Obrova et al., 2022). Importantly, NTP enables the safe reuse of sensitive items, which may reduce waste and mitigate contamination risks during periods of high viral circulation or supply shortages, as highlighted during recent pandemic conditions (Obrova et al., 2022; Scholtz et al., 2023), making it a promising disinfection technology capable of addressing longstanding challenges in managing virus-contaminated porous materials.

## Data Availability

The original contributions presented in the study are included in the article/Supplementary material, further inquiries can be directed to the corresponding author.
