# Supplementary material for: Non-thermal plasma efficiently decontaminates sensitive materials from respiratory viruses
Source: Front Microbiol. 2026 Sep 24;17:1897956. doi: 10.3389/fmicb.2026.1897956 (PMC13646080; doi:10.3389/fmicb.2026.1897956)
Supplement: Supplementary file 1 [file Data_Sheet_1.DOCX]

Supplementary Material

# 1 Supplementary Methods

**Assessment of material toxicity**

To evaluate whether the materials themselves affected viral infectivity or cell viability we used HRV and HAdV, and HeLa and A549 cells. Paper, plush fabric and foam rubber were soaked directly in 1 mL virus suspension in culture medium displaying a median tissue culture infectious dose of 10^6^ IU/mL and subjected to 5 times 5 sec vortexing. As a negative control, an identical virus suspension, where none of the materials were soaked, was used. Virus infectious titer determination was performed as described below. The same was done for assessing potential toxic effects on the cells by soaking paper, plush fabric and foam rubber in 1 mL culture medium and the extract pipetted on the cells.

# 2 Supplementary Results

**Materials do not influence viral infectivity and cell viability**

When HRV and HAdV were exposed to paper samples, only a slight, statistically non‑significant reduction in infectivity was observed—most notable for HRV (81.7±12.7% infectivity, *p*=0.38) and minimal for HAdV (96.1±8.4% infectivity, *p*=0.42) compared to the control, which did not come in contact with the materials (Supplemental Figure 1). Plush fabric produced a somewhat stronger, yet still non-significant, decline in infectivity (HRV: 64.8±15.7% (*p*=0.29); HAdV 72.7±21.9% (*p*=0.33)). However, in both cases it failed to reach statistical significance (Supplemental Figure 1). For foam rubber, HRV infectivity remained largely unchanged (97.5±39.5% infectivity, *p*=0.42), while HAdV infectivity was unaffected (115.7±19.3% infectivity, *p*=0.42), and without any statistical significance (Supplemental Figure 1). These findings indicate that none of the tested materials exerted an inhibitory or enhancing effect on viral infectivity.

When HeLa (Supplemental Figure 2) and A549 cells (Supplemental Figure 3) were exposed to extracts from paper, plush fabric, or foam rubber, no detectable cytotoxic effects were observed compared with untreated controls.

Thus, none of the materials used in this study interfered with cell viability or contributed to false-positive reductions in infectious titer.

# 3 Supplementary Figures

**Supplementary Figure 1.** Effect of the materials tested (paper, plush fabric and foam rubber) on the infectivity of HRV and HAdV.

**Supplemental Figure 2:** Effect of the materials tested (paper, plush fabric and foam rubber) on the viability of HeLa cells.

**Supplemental Figure 3:** Effect of the materials tested (paper, plush fabric and foam rubber) on the viability of A549 cell
